# The impact of COVID-19 vaccination on long-term risk of new-onset atrial fibrillation/flutter after COVID-19 infection: A retrospective cohort study

**DOI:** 10.1371/journal.pone.0348133

**Published:** 2026-04-24

**Authors:** Ching-Chung Ko, Jheng-Yan Wu, Kuo-Chuan Hung, Shu-Wei Liao, Ya-Wen Tsai, Tsung Yu, Chien-Ming Lin, I-Wen Chen

**Affiliations:** 1 Department of Medical Imaging, Chi Mei Medical Center, Tainan, Taiwan; 2 Department of Health and Nutrition, Chia Nan University of Pharmacy and Science, Tainan, Taiwan; 3 School of Medicine, College of Medicine, National Sun Yat-Sen University, Kaohsiung, Taiwan; 4 Department of Nutrition, Chi Mei Medical Center, Tainan City, Taiwan; 5 Department of Public Health, College of Medicine, National Cheng Kung University, Tainan, Taiwan; 6 Department of Anesthesiology, Chi Mei Medical Center, Tainan City, Taiwan; 7 Center of General Education, Chia Nan University of Pharmacy and Science, Tainan, Taiwan; 8 Division of Preventive Medicine, Chi Mei Medical Center, Tainan, Taiwan; 9 Department of Anesthesiology, Chi Mei Medical Center, Liouying, Tainan City, Taiwan; University of Naples Federico II: Universita degli Studi di Napoli Federico II, ITALY

## Abstract

**Purpose:**

COVID-19 infection has been associated with cardiovascular complications, including new-onset atrial fibrillation/flutter (NOAF). However, the potential protective effect of COVID-19 vaccination against long-term NOAF risk following COVID-19 infection remains unclear.

**Methods:**

This retrospective cohort study used the TriNetX Research Network to identify adults diagnosed with COVID-19. Patients were divided into a vaccine group and control group (unvaccinated). After propensity score matching (238,750 patients per group), we assessed the primary outcome of 24-month NOAF incidence, with secondary outcomes at 1, 6 and 12 months. Subgroup analyses examined effects across patient characteristics and comorbidities. Sensitivity analysis was performed by excluding patients with severe COVID-19 illness.

**Results:**

The 24-month NOAF incidence was significantly lower in the vaccine group compared to the control group (1.91% vs 2.18%; HR: 0.82, 95% CI: 0.78–0.85). This protective effect was also observed at 1 month (HR: 0.73, p < 0.001), 6 months (HR: 0.71, p < 0.001), and 12 months (HR: 0.77, p < 0.001). Sensitivity analysis confirmed these findings (HR: 0.79 at 24 months). Subgroup analyses demonstrated that COVID-19 vaccination provided significant protection against NOAF across all examined subgroups, with younger patients (18–60 years) showing greater risk reduction compared to older individuals.

**Conclusion:**

COVID-19 vaccination was associated with a significantly reduced 24-month risk of NOAF after COVID-19 infection. These findings suggest vaccination may mitigate long-term cardiovascular sequelae of COVID-19. Future research should elucidate underlying protective mechanisms and optimize vaccination strategies for cardiovascular protection, particularly in high-risk populations.

## 1. Introduction

While respiratory symptoms are the most common manifestation of COVID-19, growing evidence suggests that patients can also experience cardiovascular complications, including myocardial injury, arrhythmias, and thromboembolism [1–4]. In the short term, several studies have reported an increased incidence of new-onset atrial fibrillation/flutter (NOAF) in patients with COVID-19 [5,6], with a meta-analysis of 31 studies indicating an 8% occurrence rate [7]. The underlying mechanisms are believed to include systemic inflammation, hypoxia, and autonomic nervous system alterations [8,9]. NOAF is associated with an increased risk of complications and mortality in COVID-19 patients [10–12]. In the long term, COVID-19 infection can also lead to significant cardiovascular sequelae. A recent meta-analysis, involving approximately 20 million individuals, found that those who have recovered from COVID-19 have a higher risk of developing NOAF compared to the general population [13]. During a mean follow-up of 14.5 months, 2.6% of patients may develop NOAF after recovering from COVID-19, with the risk being notably higher among older adults, men, and individuals with pre-existing cardiovascular conditions [13]. Although COVID-19 primarily affects the respiratory system, the pulmonary inflammation, hypoxemia, and systemic cytokine activation induced by SARS-CoV-2 infection can exert substantial stress on the cardiovascular system. These processes may increase atrial pressure and promote electrical and structural remodeling, providing a biological basis for the development of NOAF following COVID-19 infection.

COVID-19 vaccines have been developed and administered to millions of people worldwide to control the pandemic [14]. These vaccines have demonstrated high efficacy in preventing symptomatic COVID-19 and reducing the risk of severe illness and hospitalization [15–17]. In addition to its short-term effect, evidence showed that the COVID-19 vaccine was associated with a significant protective effect against the development of long COVID [18,19]. This suggests that vaccination not only helps in preventing the acute phase of the disease but also plays a crucial role in reducing the risk of prolonged symptoms and complications related to the COVID-19. NOAF poses a significant public health challenge due to its association with a heightened risk of stroke, systemic embolism, and heart failure [20,21]. To the best of our knowledge, the effect of COVID-19 vaccination on the long-term NOAF risk after COVID-19 infection remains unclear. Understanding their relationship could have important implications for the management and prevention of cardiovascular complications in COVID-19 patients. This retrospective cohort study aimed to investigate the potential beneficial effect of COVID-19 vaccination on reducing the long-term risk of NOAF in patients after COVID-19 recovery.

## 2. Method

### 2.1. Data source and ethics statement

TriNetX is a global federated health research network that provides real-time updates of anonymized electronic medical records (EMRs), primarily from the United States. To ensure compliance with legal and ethical standards that prevent data re-identification, the identities of participating HCOs and their individual data contributions were kept confidential. All data provided by TriNetX are fully de-identified in accordance with HIPAA standards, and no author had access to personally identifiable information during or after data collection.

The study was conducted in accordance with the principles of the Declaration of Helsinki and was approved by the Institutional Review Board of Chi Mei Medical Center (IRB No. 11302-E01), which granted a waiver of informed consent. The data used in this retrospective study were accessed through the TriNetX Research Network on September 01, 2024. This cohort study was reported in accordance with the STROBE (Strengthening the Reporting of Observational Studies in Epidemiology) guidelines.

### 2.2. Study population

Adult patients aged 18 years or older with a first recorded diagnosis of COVID-19 between January 1, 2022 and June 30, 2023 and with more than two healthcare organization (HCO) visits were considered eligible. The date of first COVID-19 diagnosis was defined as the index date. Patients were assigned to the vaccine group if they had received at least one dose of a COVID-19 vaccine at least 14 days before the index date, thereby ensuring that vaccination preceded infection. Patients were assigned to the control group if they had no recorded COVID-19 vaccination before or after the index date, in order to maintain a clearly defined unvaccinated cohort and avoid exposure misclassification related to post-index vaccination.

To ensure the assessment of incident atrial fibrillation/flutter and improve cohort comparability, patients were excluded from both groups if they had any of the following before the index date: a history of heart transplant, cardiac surgery, pacemaker implantation, ventricular arrhythmias, atrial fibrillation, or atrial flutter. Patients who died within 24 months after the index date were also excluded to ensure comparable follow-up opportunity for outcome ascertainment across study groups.

### 2.3. Data extraction and definition

Demographic variables included age at index date, sex, and race. Baseline comorbidities were identified using International Classification of Diseases, Tenth Revision, Clinical Modification (ICD-10-CM) diagnosis codes recorded within 3 years before the index date. These comorbidities included overweight and obesity, malnutrition, alcohol-related disorders, nicotine dependence, diabetes mellitus, neoplasms, essential hypertension, dyslipidemia, chronic lower respiratory diseases, chronic kidney disease, liver diseases, cerebrovascular diseases, ischemic heart diseases, and heart failure. Available laboratory data recorded within 3 years before the index date were also extracted, including hemoglobin, estimated glomerular filtration rate (eGFR), serum albumin, and hemoglobin A1c. These baseline variables were used to characterize the study population and were included in the propensity score matching procedure.

### 2.4. Outcomes

The primary outcome was incident NOAF after COVID-19 infection. NOAF was defined by relevant ICD-10-CM diagnosis codes recorded after the index date in patients without a prior history of atrial fibrillation or atrial flutter. For the primary analysis, follow-up began 30 days after the index date to reduce the influence of acute arrhythmic events occurring during the early phase of COVID-19 infection. Patients were then followed for outcome occurrence from 1 month to 24 months after the index date. To further evaluate the temporal pattern of risk, secondary analyses were performed across prespecified time intervals, including within 1 month, 1–6 months, and 1–12 months, after the index date.

### 2.5. Sensitivity analysis

To address potential residual confounding related to COVID-19 illness severity, we conducted a sensitivity analysis that excluded patients who experienced sepsis, required hospitalization, intensive care unit (ICU) admission, or intubation within one month after COVID-19 infection. This additional analysis aimed to ensure that the observed association between vaccination and NOAF was not driven by differences in disease severity.

### 2.6. Validation analysis: COVID-19 vs. acute upper respiratory infection

To validate the association between COVID-19 lung infection and NOAF, an additional comparative analysis was performed using a separate cohort of unvaccinated patients. Using the same exclusion criteria, adult patients with a first diagnosis of COVID-19 infection or acute upper respiratory infection (AURI) between January 2022 and June 2023 were identified from the TriNetX Research Network. Those with any previous record of COVID-19 vaccination, or pre-existing atrial fibrillation/flutter were excluded in both groups. Patients diagnosed with COVID-19 infection constituted the exposure group, while those with AURI served as the reference group. Both cohorts were matched 1:1 using propensity scores derived from baseline demographic characteristics, comorbidities, and laboratory parameters, consistent with the matching strategy in the primary analysis. The primary outcome was the cumulative incidence of NOAF during 2-year follow-up.

### 2.8. Statistical analyses

To reduce the influence of confounding variables, we used propensity score matching to ensure that the vaccine and control groups had similar baseline characteristics and comorbidities. This process involved using the built-in function of the TriNetX platform to perform 1:1 matching between the two groups, employing a greedy nearest-neighbor algorithm. The matching criteria included key factors, such as age at the index date, sex, race, body mass index (BMI), and existing comorbidities, ensuring that the groups were as comparable as possible. To evaluate the effectiveness of the matching process, we calculated the standardized mean difference for each baseline characteristic. A standardized difference of less than 0.1 was considered to indicate a negligible difference between the groups, suggesting that the matching was successful in balancing the characteristics across the vaccine and control groups. Propensity score distribution was visualized to assess the overlap and adequacy of matching between the vaccinated and control cohorts. The distribution curves were plotted before and after matching to confirm successful covariate balance.

During this period, we calculated the hazard ratio (HR) for the cumulative incidence of NOAF in both the vaccine and control groups. All statistical analyses were conducted with a 95% confidence interval (95% CI), which was used to determine the statistical significance of the results. To further analyze survival probabilities over time, we employed the Kaplan-Meier method, a widely used technique in survival analysis. Subgroup analyses were conducted at the 24-month follow-up to explore the benefits of COVID-19 vaccination across various patient populations. These subgroups included sex, age (18–60 years vs. ≥ 60 years), vaccination status (1st dose, 2nd dose, ≥ 3rd dose), and comorbidities (e.g., presence of coronary artery disease). Statistical significance in this context was defined as a p-value less than 0.05, indicating that the observed differences were unlikely to be due to chance.

## 3. Results

### 3.1. Patient selection

**Fig 1:**
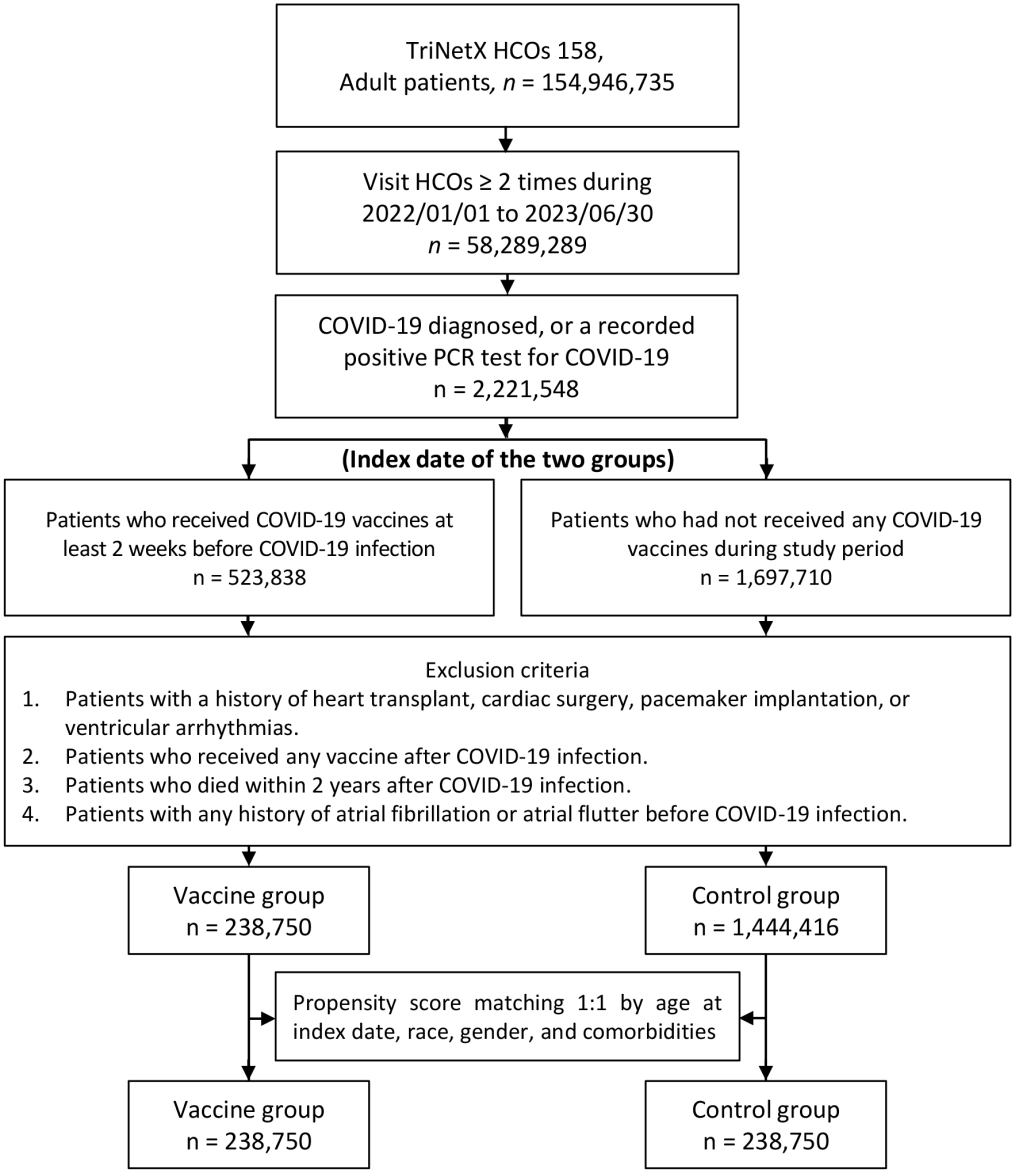
Flow chart for identification of patients with or without COVID-19 vaccination.

**Fig 2:**
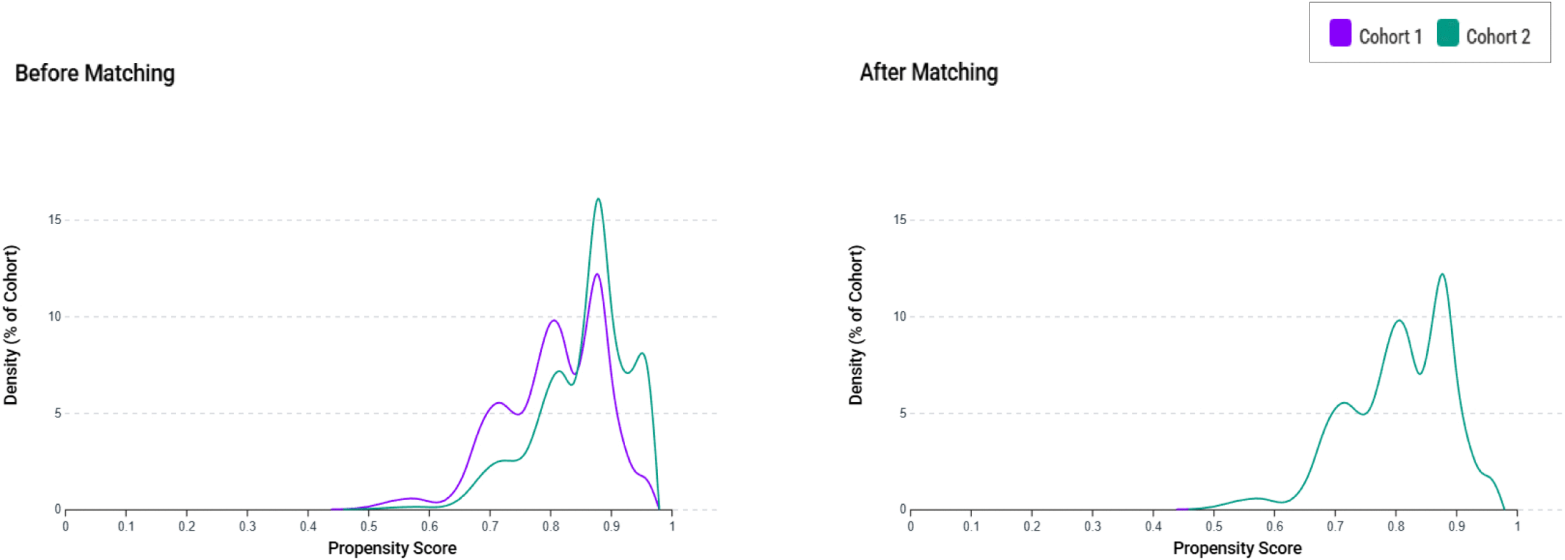
Propensity score density plots before and after matching. Density distributions of propensity scores for vaccine group (Cohort 1, purple) and control group (Cohort 2, green) are shown before matching (left panel) and after 1:1 propensity score matching (right panel). Prior to matching, the two cohorts exhibited noticeable differences in score distributions, indicating baseline imbalance. After matching, the distributions demonstrated substantial overlap, confirming improved covariate balance between groups and appropriateness of the matched analytic sample.

Fig 1 illustrates the selection of the study population from 158 HCOs in the TriNetX database. Initially, a total of 2,221,548 adult individuals diagnosed with COVID-19 or who tested positive via PCR were identified. Among them, 523,838 received vaccines at least two weeks prior to COVID-19 infection, while 1,697,710 remained unvaccinated during the study period. After the application of exclusion criteria, the subsequent analysis of the study population narrowed down to a vaccine group comprising 238,750 individuals. Additionally, a control group consisting of 1,444,416 individuals was included for comparison. Propensity score matching was then employed to create a balanced comparison, resulting in a matched vaccine group and control group, both consisting of 238,750 individuals. As illustrated in Fig 2, the distributions of propensity scores between the vaccinated and control groups showed substantial overlap after matching, confirming that baseline covariates were well balanced across cohorts.

### 3.2. Patient characteristics

Tables 1 and S1 summarizes the variables in the study population before and after matching in the vaccine and control groups. The mean age at the index date for the vaccine group was 52.5 years, while in the control group, the mean age was 48.4 years before matching, and 52.3 years after matching. In terms of sex, the majority of individuals in both groups were female, with 62.4% in the vaccine group and 62.9% in the control group. The mean BMI was 29.5 kg/m² in the vaccine group and 29.9 kg/m² in the control group before matching, and 29.4 kg/m² and 29.5 kg/m² after matching. Among the study population, the most common diseases were hypertension (vaccine vs. control group: 33.8% vs. 22.6% before matching, 33.8% vs. 33.3% after matching) and dyslipidemia (vaccine vs. control group: 38.2% vs. 21.3% before matching, 38.2% vs. 37.4% after matching). After matching, the differences in age, sex, race, BMI, and comorbidities between the two groups were small and well-matched (Table 1).

**Table 1:** Characteristics of patients and comorbidities before and after matching.

Variables^†^	Before matching			After matching		
	Vaccine group (n = 238,750)	Control group (n = 1,444,416)	SMD*	Vaccine group (n = 238,750)	Control group (n = 238,750)	SMD*
Age at index date (years)	52.5 ± 18.6	48.4 ± 19.4	0.215	52.5 ± 18.6	52.3 ± 18.6	0.014
Female	148934 (62.4)	879630 (60.9)	0.030	148934 (62.4)	150193 (62.9)	0.011
Body mass index (kg/m^2^)	29.5 ± 7.2	29.9 ± 7.6	0.054	29.4 ± 7.2	29.5 ± 7.4	0.008
White	165776 (69.4)	824604 (57.1)	0.258	165776 (69.4)	166402 (69.7)	0.006
Black or African American	25261 (10.6)	201860 (14.0)	0.104	25261 (10.6)	24807 (10.4)	0.006
Asian	22546 (9.4)	60702 (4.2)	0.209	22546 (9.4)	22456 (9.4)	0.001
Unknown Race	13774 (5.8)	278491 (19.3)	0.417	13774 (5.8)	13491 (5.7)	0.005
Comorbidities, n (%)						
Dyslipidemia	91251 (38.2)	307589 (21.3)	0.377	91251 (38.2)	89352 (37.4)	0.016
Essential hypertension	80669 (33.8)	326161 (22.6)	0.251	80669 (33.8)	79529 (33.3)	0.010
Neoplasms	54062 (22.6)	218649 (15.1)	0.193	54062 (22.6)	53654 (22.5)	0.004
Overweight and obesity	42401 (17.8)	186099 (12.9)	0.136	42401 (17.8)	41344 (17.3)	0.012
Chronic lower respiratory diseases	36062 (15.1)	177900 (12.3)	0.081	36062 (15.1)	35992 (15.1)	0.001
Diabetes mellitus	31742 (13.3)	152850 (10.6)	0.084	31742 (13.3)	30214 (12.7)	0.019
Ischemic heart diseases	17589 (7.4)	80493 (5.6)	0.073	17589 (7.4)	16226 (6.8)	0.022
Nicotine dependence	14155 (5.9)	105094 (7.3)	0.054	14155 (5.9)	13520 (5.7)	0.011
Chronic kidney disease (CKD)	14107 (5.9)	63337 (4.4)	0.069	14107 (5.9)	12551 (5.3)	0.028
Diseases of liver	13571 (5.7)	60654 (4.2)	0.069	13571 (5.7)	12390 (5.2)	0.022
Cerebrovascular diseases	8887 (3.7)	48218 (3.3)	0.021	8887 (3.7)	8192 (3.4)	0.016
Heart failure	4700 (2.0)	34222 (2.4)	0.028	4700 (2.0)	4031 (1.7)	0.021
Alcohol related disorders	3917 (1.6)	26020 (1.8)	0.012	3917 (1.6)	3606 (1.5)	0.010
Malnutrition	2025 (0.8)	11602 (0.8)	0.005	2025 (0.8)	1652 (0.7)	0.018
Laboratory data						
Hemoglobin ≥ 12 g/dL	140009 (58.6)	650650 (45.0)	0.275	140009 (58.6)	140477 (58.8)	0.004
eGFR ≥ 60 mL/min/1.73 m²	141002 (59.1)	635133 (44.0)	0.305	141002 (59.1)	142063 (59.5)	0.009
Albumin ≥3.5 g/dL	145210 (60.8)	600011 (41.5)	0.393	145210 (60.8)	144508 (60.5)	0.006
Hemoglobin A1c≥9%	1379 (0.6)	8344 (0.6)	0.000	1379 (0.6)	1160 (0.5)	0.013

^†^Data is presented as mean ± SD or number (proportion); eGFR: Estimated Glomerular Filtration Rate; SMD: standardized mean difference; * < 0.1 is considered as a small difference.

### 3.3. Primary and secondary outcomes

**Fig 3:**
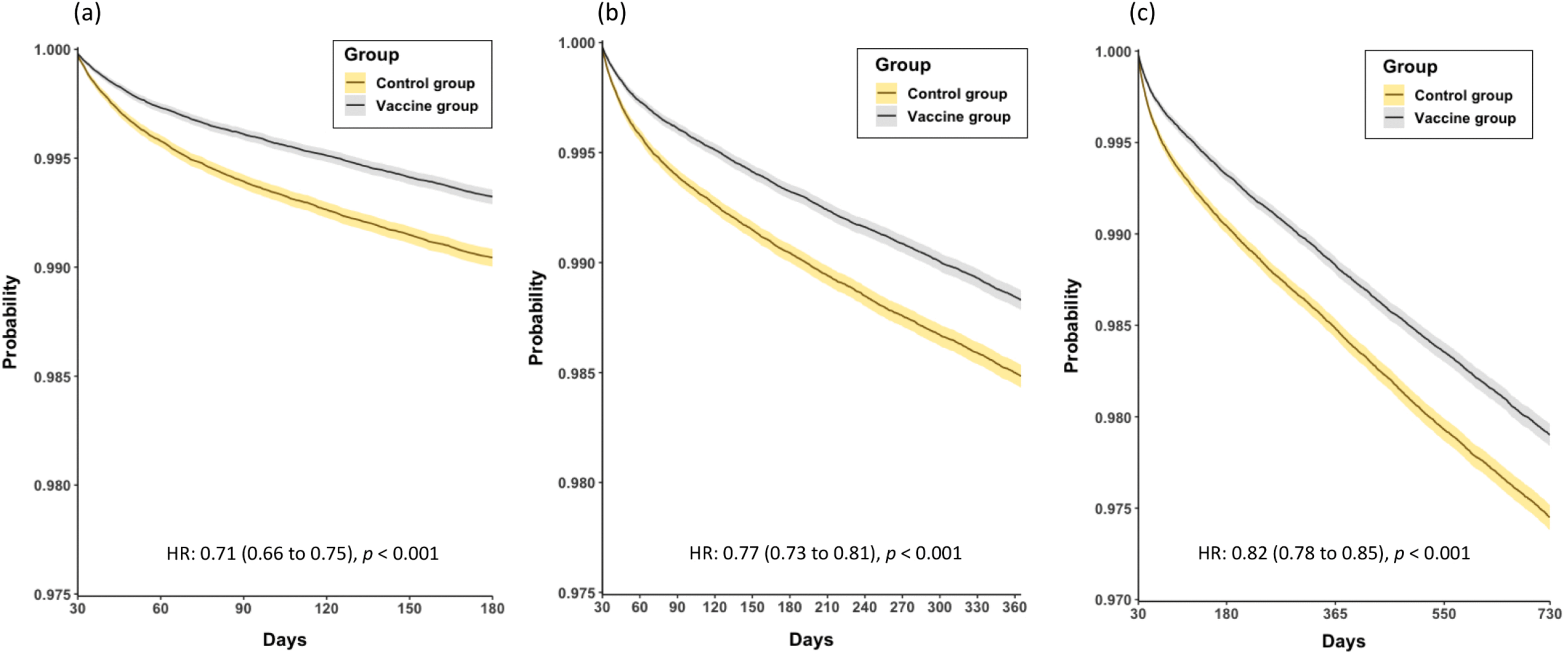
Incidence of new-onset atrial fibrillation/flutter (NOAF) in patients with or without previous COVID-19 vaccination at (a) 6-month (HR: 0.71, 95% CI: 0.66–0.75); (b) 12-month (HR: 0.77, 95% CI: 0.73–0.81), and (c) 24-month (HR: 0.82, 95% CI: 0.78–0.85) follow-ups. HR: hazard ratio.

Table 2 presents the risk of NOAF in patients with or without previous COVID-19 vaccination across different follow-up periods. In both groups, the number of patients with NOAF increased with time. Across sequential follow-up intervals, vaccinated individuals consistently demonstrated lower event rates than controls. From the index date to 1 month, incidence was 0.44% versus 0.59% (HR 0.73, 95% CI 0.67–0.79; p < 0.001). Between 1 and 6 months, incidence was 0.64% versus 0.88% (HR 0.71, 95% CI 0.66–0.75; p < 0.001). At 1–12 months, incidence reached 1.10% versus 1.37% (HR 0.77, 95% CI 0.73–0.81; p < 0.001). For the primary 1–24-month outcome window, cumulative incidence was 1.91% versus 2.18% (HR 0.82, 95% CI 0.78–0.85; p < 0.001). Patients with previous COVID-19 vaccination had a significantly lower risk of NOAF across all long-term follow-up intervals (Fig 3a-c).

**Table 2:** Risk of new-onset atrial fibrillation/flutter in patients with or without previous COVID-19 vaccination.

NOAF Follow-up period (months)	COVID-19 survivors		HR (95% CI)	*P* value
	Vaccine group (N = 238,750)	Control group (N = 238,750)		
Index date-1m	1,042 (0.44%)	1,416 (0.59%)	0.73 (0.67-0.79)	< 0.001
1-6m	1,532 (0.64%)	2,096 (0.88%)	0.71 (0.66-0.75)	< 0.001
1-12m	2,616 (1.10%)	3,261 (1.37%)	0.77 (0.73-0.81)	< 0.001
1-24m	4,547 (1.91%)	5,211 (2.18%)	0.82 (0.78-0.85)	<0.001

NOAF: new-onset atrial fibrillation/flutter; HR: Hazard ratio; CI: confidence interval.

### 3.4. Sensitivity analysis

As shown in Table 3, after excluding patients who experienced sepsis, required hospitalization, ICU admission, or intubation within one month after COVID-19 infection, the protective association between COVID-19 vaccination and NOAF remained consistent across all follow-up intervals. In a sensitivity cohort comprising 218,133 matched pairs, the protective association of vaccination persisted across all examined durations. The vaccine group experienced consistently lower cumulative NOAF incidence, beginning in the first month (0.13% vs. 0.28%) and extending through 6 months (0.41% vs. 0.67%), 12 months (0.80% vs. 1.05%), and 24 months (1.51% vs. 1.78%). Corresponding HRs confirmed robust risk reductions at each interval (HRs 0.44, 0.60, 0.73, and 0.79; all p < 0.001). These findings suggest that the main results were robust even after excluding patients with severe or critical COVID-19 illness.

**Table 3:** Sensitivity analysis with exclusion of critically ill patients.

NOAF Follow-up period (months)	COVID-19 survivors		HR (95% CI)	*P* value
	Vaccine group (N = 218,133)	Control group (N = 218,133)		
Index date-1m	272 (0.13%)	613 (0.28%)	0.44 (0.38-0.50)	< 0.001
1-6m	904 (0.41%)	1,460 (0.67%)	0.60 (0.55-0.65)	< 0.001
1-12m	1,741 (0.8%)	2,286 (1.05%)	0.73 (0.68-0.77)	< 0.001
1-24m	3,285 (1.51%)	3,867 (1.78%)	0.79 (0.76-0.83)	<0.001

NOAF: new-onset atrial fibrillation/flutter; HR: Hazard ratio; CI: confidence interval.

### 3.5. Subgroup analysis

**Fig 4:**
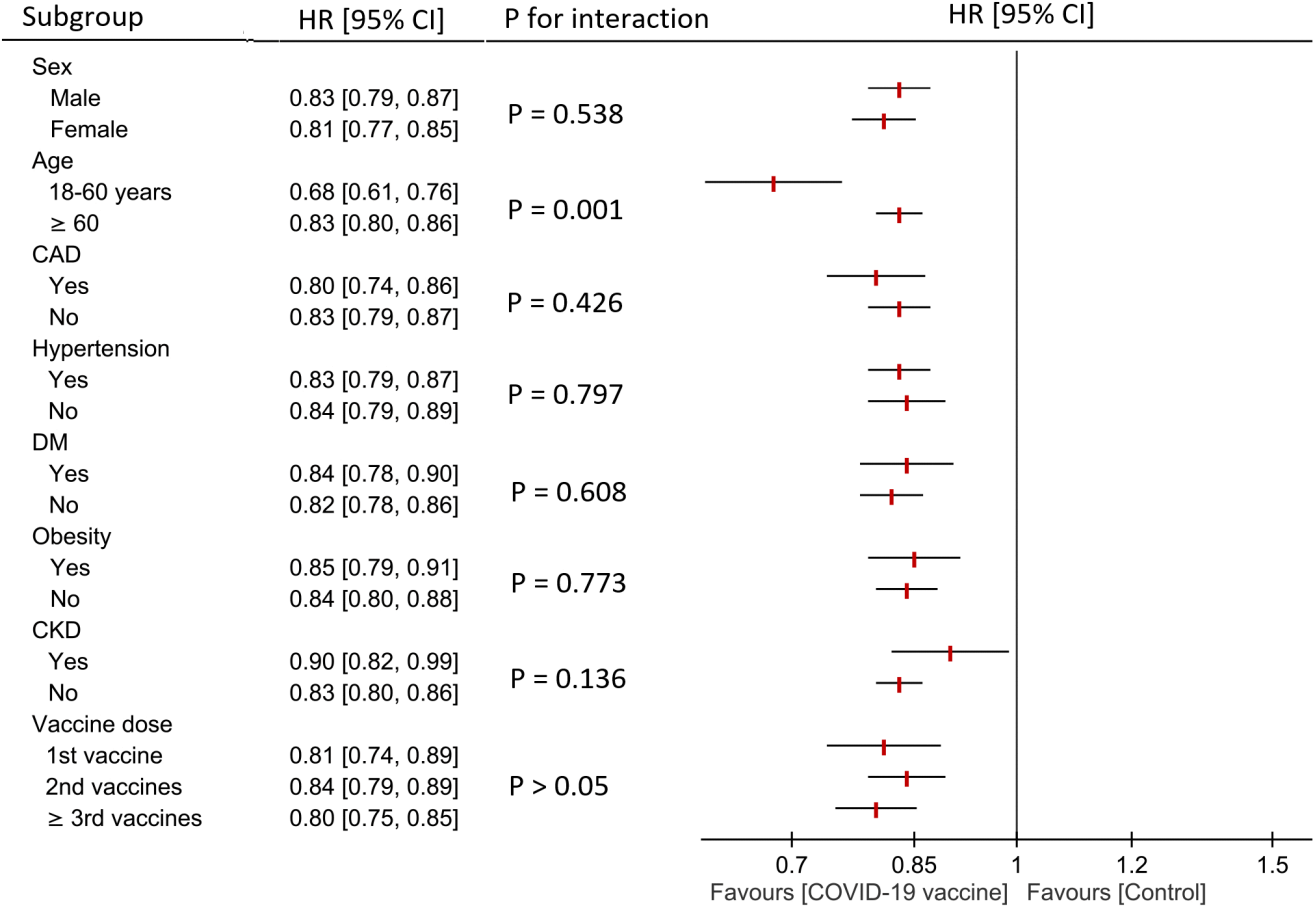
Subgroup analysis of the association between COVID-19 vaccine exposure and the risk of new-onset atrial fibrillation/flutter (NOAF) at the 24-month follow-up. Hazard ratios (HRs) and 95% confidence intervals (CIs) are presented for each subgroup, with corresponding p-values for interaction. Subgroups include sex (female, male), age (18–60 years, ≥ 60 years), coronary artery disease (CAD), obesity, diabetes mellitus (DM), hypertension (HTN), and chronic kidney disease (CKD), as well as number of vaccine doses (first dose, second dose, ≥ third dose).

Fig 4 presents the subgroup analysis of the association between COVID-19 vaccination and NOAF risk at 24-month follow-up, stratified by sex, age, comorbidities, and vaccination status. COVID-19 vaccination was associated with a significantly reduced risk of NOAF across all subgroups examined (all p < 0.05). The protective effect was observed in both female (HR: 0.81) and male patients (HR: 0.83), with no significant difference between sexes (p = 0.538). The magnitude of risk reduction was more pronounced in younger patients aged 18–60 years (HR: 0.68) compared with those aged ≥60 years (HR: 0.83; p = 0.001 for interaction). Patients with obesity, coronary artery disease, diabetes mellitus, hypertension, and chronic kidney disease all demonstrated significant protective associations with vaccination, with no significant interactions observed between these comorbidity subgroups (all p > 0.05).

### 3.5. Validation analysis

After propensity score matching, both the COVID-19 and AURI cohorts included 663,757 patients each. During follow-up, new-onset AF occurred in 9,568 patients (1.44%) in the COVID-19 group and 6,314 patients (0.95%) in the AURI group, corresponding to a HR of 1.63 (95% CI, 1.58–1.68; p < 0.0001). These findings indicate that patients with COVID-19 infection have a significantly higher risk of developing NOAF compared with those with non-COVID respiratory infections, suggesting that COVID-19 exerts a stronger cardiovascular impact beyond that of typical pulmonary infections.

## 4. Discussion

This retrospective cohort study utilizing the TriNetX global federated health research network found that COVID-19 vaccination was associated with a significantly reduced 24-month risk of NOAF following COVID-19 infection (HR: 0.82). This protective effect was consistently observed across all follow-up intervals, including the early post-infection period. Sensitivity analysis excluding patients with severe COVID-19 illness confirmed the robustness of these findings. Validation analysis demonstrated that COVID-19 infection conferred a higher NOAF risk compared with acute upper respiratory infection (HR: 1.63). Subgroup analyses revealed that vaccination provided significant protection across all examined subgroups, with younger patients (18–60 years) showing greater risk reduction than older individuals (p = 0.001 for interaction).

The precise pathophysiological mechanisms that trigger or precipitate atrial fibrillation/flutter after COVID-19 infection are not fully understood, but several potential pathways have been suggested. First, one proposed mechanism involves the binding of SARS-CoV-2 to angiotensin-converting enzyme 2 (ACE2) receptors, which are expressed in myocardial microvascular pericytes. This viral interaction may lead to increased myocardial inflammation, fibrosis, tissue edema, and interstitial hydrostatic pressure, resulting in structural changes in the atria [8,22]. Second, COVID-19-induced acute respiratory distress syndrome (ARDS) and pulmonary hypertension can elevate right atrial pressure, potentially triggering atrial fibrillation/flutter [23]. Third, dysregulation of the renin-angiotensin-aldosterone system (RAAS) due to SARS-CoV-2 binding may lead to increased angiotensin II levels, which are known to promote atrial fibrosis and remodeling, further contributing to the development of atrial fibrillation/flutter [8]. Fourth, the hyperinflammatory state associated with severe COVID-19, characterized by elevated pro-inflammatory cytokines such as interleukin-6 (IL-6), may also directly affect atrial myocytes and promote atrial fibrillation/flutter [8,13,24]. Finally, COVID-19 may cause autonomic dysfunction, leading to increased sympathetic activity and decreased heart rate variability, which could trigger atrial fibrillation/flutter in susceptible individuals [8,13,24]. Taken together, these mechanisms suggest that the severity of infection and the magnitude of the associated inflammatory and cardiopulmonary stress may play an important role in post-COVID atrial arrhythmogenesis. Accordingly, COVID-19 vaccination may reduce the risk of NOAF indirectly by attenuating infection severity and the downstream inflammatory, hemodynamic, and autonomic disturbances that promote atrial electrical instability.

Our finding that COVID-19 vaccination is associated with a reduced risk of NOAF adds to the growing body of evidence that vaccination may have a protective effect against the development of post-acute sequelae of COVID-19. Several systematic reviews and meta-analyses found that individuals who received COVID-19 vaccination prior to COVID-19 infection had a lower risk of developing long COVID compared to those who were unvaccinated, particularly in patients who received two or more doses [18,19,25]. The mechanisms by which COVID-19 vaccines may prevent or alleviate long COVID are not fully understood, but several potential mechanisms are possible [24]. First, vaccination may attenuate the immune responses and inflammation that contribute to persistent symptoms [24]. Second, by reducing the severity of acute COVID-19 illness, vaccination could lower the risk of organ damage and the development of persistent symptoms associated with long COVID [24]. Our findings, alongside existing research, highlight the potential of COVID-19 vaccines not only to prevent acute illness but also to reduce the long-term cardiovascular impacts of SARS-CoV-2 infection.

Our study found that COVID-19 vaccination provided significant protection against NOAF across all examined subgroups. Notably, younger patients aged 18–60 years demonstrated greater risk reduction (HR: 0.68) compared with older patients aged ≥60 years (HR: 0.83, p = 0.001 for interaction), suggesting that age may modify the protective effect of vaccination. This finding is clinically relevant, as older adults generally face higher baseline cardiovascular risk and may benefit from additional preventive strategies beyond vaccination. The protective effect was consistently observed in patients both with and without major comorbidities, indicating that the cardioprotective benefits of COVID-19 vaccination extend broadly across diverse patient populations, regardless of comorbidity burden. However, the presence of these conditions did not significantly alter the magnitude of vaccine-associated risk reduction, highlighting that while vaccination provides protection, individuals with these comorbidities remain at elevated absolute risk and may require intensified cardiovascular monitoring and management following COVID-19 infection.

Our findings have important implications for clinical practice, public health policies, and future research. These results underscore the importance of promoting COVID-19 vaccination among patients, not only to prevent acute illness, but also to potentially reduce the risk of long-term cardiovascular complications such as NOAF. While the absolute difference between groups was relatively small, the observed association may still be clinically meaningful because atrial fibrillation/flutter is linked to serious downstream consequences, including stroke, heart failure, and recurrent healthcare use. At the population level, even a modest reduction in NOAF could have meaningful clinical implications, especially in individuals at higher cardiovascular risk. In terms of policy, this study supports the continuation and expansion of efforts to improve vaccine uptake, as widespread immunization may have broader health benefits beyond the immediate pandemic response. Future research should focus on elucidating the underlying mechanisms of the protective effect of vaccination against NOAF, such as investigating its impact on inflammatory pathways, thrombotic risk, and cardiac remodeling.

This study has several limitations that should be considered when interpreting the results. First, the retrospective design relies on the accuracy and completeness of electronic medical records, which may be subject to coding errors or missing data. The identification of NOAF in this study was based solely on diagnostic codes within the TriNetX network; therefore, asymptomatic or paroxysmal atrial fibrillation or atrial flutter cases diagnosed outside participating institutions may have been under detected, potentially leading to an underestimation of the true incidence. Second, although propensity score matching was used to balance baseline characteristics between the vaccinated and unvaccinated cohorts, there remains a potential for residual confounding by unmeasured factors, such as socioeconomic status, lifestyle habits, and healthcare access. Third, the study did not have detailed information on COVID-19 severity, inflammatory markers, or cardiac function, which could have influenced the risk of NOAF and protective effect of vaccination. Fourth, data on the specific types of COVID-19 vaccines received by patients were not available, precluding comparisons between different vaccine platforms or manufacturers. Finally, the study population was predominantly based in the United States, and the findings may not be generalizable to other geographic regions or healthcare systems with different patient characteristics, vaccination policies, or COVID-19 epidemiology. In addition, this study did not include comparisons with other respiratory infection vaccines, such as influenza or pneumococcal vaccines, to validate whether the observed cardioprotective association is specific to the COVID-19 vaccine. Future research incorporating these vaccine types would help determine whether the reduction in NOAF risk reflects a general effect of respiratory vaccination or a unique feature of COVID-19 immunization. Despite these limitations, the large sample size and the use of a propensity score matching approach enhanced the validity of the observed association between COVID-19 vaccination and a reduced risk of NOAF.

## 5. Conclusions

In conclusion, COVID-19 vaccination was associated with a significantly reduced 24-month risk of NOAF following COVID-19 infection. Sensitivity analysis excluding severe COVID-19 cases confirmed these findings. These results suggest that COVID-19 vaccination may offer cardioprotective benefits beyond preventing acute illness, potentially mitigating long-term cardiovascular sequelae. Future studies should elucidate underlying mechanisms and optimize vaccination strategies for cardiovascular protection, particularly through randomized controlled trials.

## Supporting information

S1 Table Baseline characteristics and comorbidities of the vaccine and control cohorts before and after propensity score matching, with corresponding p-values. (DOCX)
