# Supplementary material for: The impact of COVID-19 vaccination on long-term risk of new-onset atrial fibrillation/flutter after COVID-19 infection: A retrospective cohort study
Source: PLoS One. 2026 Apr 24;21(4):e0348133. doi: 10.1371/journal.pone.0348133 (PMC13108787; doi:10.1371/journal.pone.0348133)
Supplement: S1 Table — (DOCX) [file pone.0348133.s001.docx]

**Supplemental table 1.** Baseline characteristics and comorbidities of the vaccine and control cohorts before and after propensity score matching, with corresponding p-values

| Variables† | Before matching | | |  | After matching | | |
| --- | --- | --- | --- | --- | --- | --- | --- |
|  | Vaccine group  (n= 238,750) | Control group  (n= 1,444,416) | P-value | Vaccine group  (n= 238,750) | | Control group  (n= 238,750) | P-value |
| Age at index date (years) | 52.5 ± 18.6 | 48.4 ± 19.4 | <0.001 | 52.5 ± 18.6 | | 52.3 ± 18.6 | <0.001 |
| Female | 148934 (62.4) | 879630 (60.9) | <0.001 | 148934 (62.4) | | 150193 (62.9) | 0.011 |
| Body mass index (kg/m^2^) | 29.5 ± 7.2 | 29.9 ± 7.6 | <0.001 | 29.4 ± 7.2 | | 29.5 ± 7.4 | 0.026 |
| White | 165776 (69.4) | 824604 (57.1) | <0.001 | 165776 (69.4) | | 166402 (69.7) | 0.049 |
| Black or African American | 25261 (10.6) | 201860 (14.0) | <0.001 | 25261 (10.6) | | 24807 (10.4) | 0.032 |
| Asian | 22546 (9.4) | 60702 (4.2) | <0.001 | 22546 (9.4) | | 22456 (9.4) | 0.656 |
| Unknown Race | 13774 (5.8) | 278491 (19.3) | <0.001 | 13774 (5.8) | | 13491 (5.7) | 0.078 |
| Comorbidities, n (%) |  |  |  |  | |  |  |
| Dyslipidemia | 91251 (38.2) | 307589 (21.3) | <0.001 | 91251 (38.2) | | 89352 (37.4) | <0.001 |
| Essential hypertension | 80669 (33.8) | 326161 (22.6) | <0.001 | 80669 (33.8) | | 79529 (33.3) | <0.001 |
| Neoplasms | 54062 (22.6) | 218649 (15.1) | <0.001 | 54062 (22.6) | | 53654 (22.5) | 0.158 |
| Overweight and obesity | 42401 (17.8) | 186099 (12.9) | <0.001 | 42401 (17.8) | | 41344 (17.3) | <0.001 |
| Chronic lower respiratory diseases | 36062 (15.1) | 177900 (12.3) | <0.001 | 36062 (15.1) | | 35992 (15.1) | 0.777 |
| Diabetes mellitus | 31742 (13.3) | 152850 (10.6) | <0.001 | 31742 (13.3) | | 30214 (12.7) | <0.001 |
| Ischemic heart diseases | 17589 (7.4) | 80493 (5.6) | <0.001 | 17589 (7.4) | | 16226 (6.8) | <0.001 |
| Nicotine dependence | 14155 (5.9) | 105094 (7.3) | <0.001 | 14155 (5.9) | | 13520 (5.7) | <0.001 |
| Chronic kidney disease (CKD) | 14107 (5.9) | 63337 (4.4) | <0.001 | 14107 (5.9) | | 12551 (5.3) | <0.001 |
| Diseases of liver | 13571 (5.7) | 60654 (4.2) | <0.001 | 13571 (5.7) | | 12390 (5.2) | <0.001 |
| Cerebrovascular diseases | 8887 (3.7) | 48218 (3.3) | <0.001 | 8887 (3.7) | | 8192 (3.4) | <0.001 |
| Heart failure | 4700 (2.0) | 34222 (2.4) | <0.001 | 4700 (2.0) | | 4031 (1.7) | <0.001 |
| Alcohol related disorders | 3917 (1.6) | 26020 (1.8) | <0.001 | 3917 (1.6) | | 3606 (1.5) | <0.001 |
| Malnutrition | 2025 (0.8) | 11602 (0.8) | 0.023 | 2025 (0.8) | | 1652 (0.7) | <0.001 |
| Laboratory data |  |  |  |  | |  |  |
| Hemoglobin ≥ 12 g/dL | 140009 (58.6) | 650650 (45.0) | <0.001 | 140009 (58.6) | | 140477 (58.8) | 0.169 |
| eGFR≥60 mL/min/1.73 m² | 141002 (59.1) | 635133 (44.0) | <0.001 | 141002 (59.1) | | 142063 (59.5) | 0.002 |
| Albumin ≥3.5 g/dL | 145210 (60.8) | 600011 (41.5) | <0.001 | 145210 (60.8) | | 144508 (60.5) | 0.038 |
| Hemoglobin A1c≥9% | 1379 (0.6) | 8344 (0.6) | 0.996 | 1379 (0.6) | | 1160 (0.5) | <0.001 |

eGFR: Estimated Glomerular Filtration Rate
